# Assessment of barriers for people with disability to enjoy national parks

**DOI:** 10.3389/fpsyg.2022.1058647

**Published:** 2023-01-10

**Authors:** Maria José Aguilar-Carrasco, Eric Gielen, Maria Vallés-Planells, Francisco Galiana, Gabriel Riutort-Mayol

**Affiliations:** ^1^Department of Urbanism, Universitat Politècnica de València, Valencia, Spain; ^2^Department of Agrifood and Rural Engineering, Universitat Politècnica de València, Valencia, Spain; ^3^Foundation for the Promotion of Health and Biomedical Research of Valencia Region (FISABIO), Valencia, Spain

**Keywords:** people with mobility/motor disabilities (PwMDs), nature-based recreation, statistical inference, log-linear models, outdoor barriers, survey, accessibility, constraints

## Abstract

There is increasing awareness of the importance of nature-based recreation to human wellbeing. Given its significant benefits, natural-protected area management has not always provided equitable access to all potential users. Limited research till now has been focused on how the outdoor barriers impact the equal enjoyment of people with mobility/motor disabilities (PwMDs) of nature when promoting sustainable nature-based tourism. This study intends to shed light on those aspects which constrain the PwMD’s enjoyment of the great outdoors at national parks (NPs). The study was carried out in Canada and Spain through an online survey on amenities needed and accessibility barriers for PwMDs in parks. It was analyzed through log-linear models for cross-frequency tables, which allow us to estimate the associations between questions/variables and, thus, ultimately, obtain insights into how the needs of amenities and accessibility barriers can affect and limit PwMDs’ enjoyment of natural parks. The results show a predisposition on the part of participants to enjoy parks more frequently than they usually do, as well as a preference for forests and mountain landscapes. Information and communication technologies are the tools most chosen to prepare for their trip and visit to the park. PwMD finds barriers in NPs as impediments to benefit from nature-based recreation and adding to their wellbeing. NP managers should take into consideration that PwMD’s visits to parks are related to the main obstacles they perceive such as slopes and pavement and that they require amenities such as recreation and signposting. They would also like to enjoy parks more often, with footpaths suitable for walkability/wheelability, e.g., compact pavement, low inclines, and adequate signposting. In addition to the physical barriers, managers should also consider intrapersonal and interpersonal constraints to provide outdoor activities in parks focused on PwMDs’ needs.

## 1. Introduction

Nature’s enjoyment should be a right for everyone but this is not always the case (UN, 1994) since not all people have the required physical condition to accessing nature (Corazon et al., 2019). According to the WHO (2011) report on disability, around 15% of the world’s population have some type of disability and, of those, around 50% reported some mobility disabilities that affected their ability to move around or practice vigorous activity. In protected natural areas, all actions designed to improve accessibility should ensure people with disabilities (PwD) are able to fully participate in outdoor activities as defined by the International Classification of Functioning, Disability, and Health (ICF) (Crawford et al., 2008); thus, they can enjoy the same health benefits of nature as everyone else. Scholars on nature-based recreation have indirectly or directly based their studies on the benefit that humans obtain from ecosystem functions such as improvements in physical, mental, and social health, especially when they can actively participate in activities that involve enjoying green spaces (Zhang et al., 2017; Groulx et al., 2022).

Despite the consensus that exists in academia about the benefits of outdoor activities in natural spaces for human health (Van den Bosch and Ode Sang, 2017), people with mobility/motor disabilities (PwMDs) continue to encounter systematic barriers that are in one way or another preventing them from enjoying the activities under equal conditions and from the benefits that wilderness areas provide to their visitors (Burns et al., 2009). Determining the ideal conditions to promote walkability and wheelability in parks and addressing all the factors that can limit PwMDs’ involvement in nature-based recreation and tourism would be necessary to shed light on the causes that might be constraints on their going into nature. These should include what PwMDs need to know before they go (Tsai et al., 2010), what the on-site experience depends on, and which aspects might limit their outdoor experiences.

This article focuses on PwMDs in Canada and Spain and tries to provide more information about the main drawbacks highlighted as barriers they may encounter when visiting wilderness or a wilderness area. These barriers may be influencing what their final destinations are, as they might not always be the nature-based destination they would most like. To obtain insights into this, a cross-sectional statistical analysis is undertaken through an online questionnaire launched in both countries. An exploratory analysis is carried out initially to describe the data and help to formulate several hypotheses which are focused on identifying strategies that can promote or explain PwMDs’ walkability/wheelability in NPs. Finally, a statistical confirmatory analysis through log-linear models is applied to analyze and assess each one of the hypotheses (Cea and Ancona, 2001; Fagerland et al., 2017).

## 2. Nature-based recreation constraints in national parks

National park visitor numbers are increasing every year. In Canada, the Statista Research Department (2021) estimates that around 15.9 million people visited Canadian parks between March 2017 and March 2018. On the other side of the Atlantic, according to the data provided from Europarc-Spain’s statistics on 2017, one million 72 visitors visited Spanish NPs (Farías-Torbidoni et al., 2020). Considering the growing importance and the complexity of tourism in those areas, national policies must ensure nature-based tourism that is equitable for all people taking into consideration its conservation for future generations through good governance (Chikuta et al., 2021; Aguilar-Carrasco et al., 2022).

The UN (2015) General Assembly has identified universal access to safe and inclusive greenspaces as part of its sustainable cities and communities’ Sustainable Development Goal (SDG 11.7). Within this framework, tourism is one of the activities which must also be sustainable. The SDGs promote sustainable tourism, and accessibility and the inclusion of all people involved fall under these criteria (Bianchi et al., 2020; Sica et al., 2021; Sisto et al., 2022). Thereby, in alignment with the SDGs, accessibility is a challenge that should be faced in the promotion of nature-based tourism, which should contribute to the well-being of all of society (Winter et al., 2020).

The numbers for PwD in both countries are also relevant. According to the 2017 Canadian Survey on Disability (CSD), there are 6.2 million Canadians aged 15 and older living with some form of disability, of which 44.9% required at least one type of aid or assistive device (Giesbrecht et al., 2017; Choi, 2021). In Spain, the most recent State Database from the Survey of Disability, Personal Autonomy, and Situations of Dependency (Spanish Government, 2020) showed that 4.38 million citizens are considered PwD (a recognized disability of more than 33% using data for those 6 years old and over), where mobility/motor disability was the most representative cause at 54% of the total.

Williams et al. (2004) identified three types of access-to-nature constraints on PwMDs impeding them from enjoying outdoor experiences: structural, interpersonal, and intrapersonal. The first one is related to physical environment features that interfere with PwMD’s preferences and actual participation. Intrapersonal constraints are related to more psychological aspects that influence their decision to enjoy natural areas. Interpersonal constraints are related to factors that can influence people’s relationships with others like park staff or other people at parks. Previous studies (Burns and Grafe, 2007; Burns et al., 2009; Stigsdotter et al., 2017; Zhang et al., 2017; Corazon et al., 2019; Menzies et al., 2020) corroborate the relationship between mobility/motor disabilities with all these constraints, although the intrapersonal dimension has not been evaluated as thoroughly. These authors generally point out PwMD’s behavior is related to lower participation in, affordability of, and lack of availability of social groups with which to enjoy outdoor recreation. Constraints are not only visible in the enjoyment of the natural environments but are also seen when PwMDs try to be more active by practicing sports or engaging in daily activities that involve the use of open spaces in built environments such as city parks (Zhang et al., 2017; Darcy et al., 2018; Blaszczyk et al., 2020; Perry et al., 2021; Groulx et al., 2022).

It would seem that the main barriers faced by PwMDs are not only due to the physical environment itself, but also to the lack of amenities, information, distance, affordability, and social connections, all of which could be improved. Some studies begin to provide solutions, basing their central question on the relationship between green areas and accessibility for PwMD with initiatives that could better promote their inclusion in outdoor activities such as the “TrailRider programme” (James et al., 2017), accessibility in Geoparks (Henriques et al., 2018), or an app to show the accessibility characteristics of city beaches in Spain (Mayordomo-Martínez et al., 2019). NP walkability and wheelability around the park’s paths have also been evaluated (Burns et al., 2009; Menzies et al., 2020). How planning and management of parks through good governance could improve NPs’ accessibility to make them more inclusive for all people (Bianchi et al., 2020; Farías-Torbidoni et al., 2020; Groulx et al., 2021) has been described to guide park management and align with the SDGs.

According to the literature reviewed on the causes that might constrain PwMD, the focus should not just be on the physical environment but also on empowering them intrapersonally and interpersonally to use green spaces on equal terms. Therefore, having information from PwMD related to the uses of wilderness areas is the first step to complying with the international treaties (UN, 1994) signed by both countries (Canada and Spain) and their own policies.

To shed light on the assessment of barriers standing in the way of PwMDs equally enjoying NPs a set of hypotheses were formulated to closely examine causes that could be constraints on their going into wilderness spaces in Canada and Spain where, despite the growing yearly number of NP visitors, there is no data about whether people with disabilities are visiting these natural spaces.

H1. Demographic characteristics might influence the type of tools used to search for park’s information: The use of different information sources, such as emerging technology, prior knowledge, and traditional methods, to prepare for the visit might depend on the PwMDs’ demographic characteristics.

H2. The frequency of visits to a certain natural site might be determined by the perception of some elements or characteristics of the sites as barriers that may hinder or even prevent their enjoyment. This hypothesis will allow us to evaluate the perception of some elements or characteristics of the sites as barriers for PwMDs and their relationship with the frequency that PwMDs visit these sites.

H3. The frequency of visits to a certain natural site might be determined by amenities needed by PwMDs that may hinder or even prevent their enjoyment. This hypothesis will allow us to evaluate the need for some basic amenities so that PwMDs can enjoy these environments, and if the requirement of these amenities can be related to the frequency of visits.

H4. Landscape type preference might influence the green location type usually visited by PwMD. The comparison of the preferences or desires of PwMDs with the places they actually traveled to can allow us to evaluate if they can enjoy the places they really want or if they only enjoy the places that *a priori* may be more suitable for them due to their physical conditions.

The article is organized as follows: Section “3 Methodology” introduces the methodology of the research design, how the questionnaire was elaborated and distributed, and the statistical methodology used to analyze the questions. Section “4 Results” describes the results of descriptive and confirmatory statistical analysis of the data for each of the hypotheses. Section “5 Discussion” discusses and interprets the results and, finally, section “6 Conclusion” gives brief conclusions, their implications, and possible future study.

## 3. Methodology

An exploratory and confirmatory statistical study was done through an online questionnaire. It was elaborated to study the hypotheses described above. Each of the questions and their responses can be seen as variables that can be analyzed as descriptive statistics to describe the sample population and confirmatory log-linear statistical models to evaluate the association and interaction patterns among variables. The analysis and assessment of the resulting associations among variables will allow us to shed light on the needs, mobility difficulties, and desires of PwMDs when visiting NPs and to promote an equitable enjoyment of nature.

### 3.1. Questionnaire structure

The questionnaire was inspired by the existing literature in terms of the relationship between PwMD and greenspaces (Burns and Grafe, 2007; Burns et al., 2009; James et al., 2017; Stigsdotter et al., 2017; Zhang et al., 2017; Henriques et al., 2018; Corazon et al., 2019; Mayordomo-Martínez et al., 2019; Menzies et al., 2020) and the experience of one of the authors as a person with a spinal cord injury. Pre-coded questions (Cea and Ancona, 2001) were structured in two sections to organize all the information provided for PwMDs. At the beginning of the questionnaire a consent form and a description of the study objectives was included, followed by a code name question to maintain the participants’ anonymity (Government of Canada, 2000; Gobierno de España, 2014) and an age control question. The survey was only available for people over 18 because an online questionnaire could not assure minors would fill it out with parental consent.

To test the reliability of the questionnaire some rounds of qualitative evaluation were conducted to ensure the validity of each question. Attention was paid to language, the quality of images, and online functionality. The survey was conducted in Canada and Spain and distributed through an online questionnaire on the UBC-hosted version of Qualtrics (2018). Before launching the link to the audience, the study was evaluated and approved by the Behavioral Research Ethics Board of the University of British Columbia (Approval Certificate Number: H190-00951) in 2019. It was then opened to the public from 2019 to 2020. As it was an online questionnaire, the universe population was considered indeterminate because it was difficult to control how many people would answer it. We tried to give it the greatest diffusion possible to reach the maximum audience. A flyer with survey links and project information was distributed through PwMDs’ associations in both countries to promote link distribution through their network and associated people. The collected material is stored in a UBC-hosted version of the Qualtrics database.

The questionnaire is divided as follows:

• Section 1 is about the PwMD’s background. This section is composed of some questions about demographic and individual characteristics such as gender, country, age, and the type of device needed for walkability or wheelability. Additionally, two more questions were asked about driver’s licenses and whether they had access to their own car. Table 1 summarizes the variables derived from the questions asked in section 1.
• Section 2 is about the PwMDs’ use of natural locations. This section aims to determine habits and potential constraints in the use of these locations for PwMDs in terms of walkability or wheelability. In the first part, several questions were asked about the frequency, activities, and desires related to PwMDs’ use of these sites. In a second part, other questions were asked about how PwMDs perceive the distance traveled, the distance from the visitor center, the slope, the type of pavement, and the weather as potential barriers, as well as whether they consider recreational, information, education, accommodations, and parking as essential needs to visit NPs in terms of walkability or wheelability in safely outdoors (Corazon et al., 2019). Table 2 summarizes the variables derived from questions asked in section 2.

**TABLE 1:** Questionnaire’s question, variables, and categories in section 1.

Question	Variables	Categories
Location	Country and region	Canada and Spain
		Region: Canada’s provinces = 13 and Spanish’s regions^(a)^ = 19
		Clustered into two categories by country
Gender	Gender	Four categories: female, male, other, and prefer not to say
Age	Age cohorts:	Eight categories: 18–27, 28–37, 38–47, 48–57, 58–67, 68–77, > 77, prefer not to say
		Clustered into three generations: Generation Z, Millennials + Generation X, Boomers I and II, and prefer not to say
What kind of assistive device do you need?^(b)^	Assistance devices	Eight categories: crutches, walkers, manual or power wheelchair, motorized scooter, prosthesis, walking stick and canes, other
		Clustered into two groups: people who can walk with ambulation aids and people who need wheeled mobility device
Do you have a driver’s license (for a car)?	Driver’s license	Two categories: yes or no
Do you own a car?	Own car	Two categories: yes or no

^(a)^Regions in the European Union, Nomenclature of territorial units for statistics NUTS 2013/EU-28.

^(b)^International Classification of Functioning, Disability, and Health classification [(ICF), 2001] (include pictures of the assistive device) People who can walk with ambulation aids and people who need some wheeled mobility device to move (22).

**TABLE 2:** Questionnaire’s questions, their variables, and categories in section 2.

Question	Variables	Categories
What amenities are essential for you?	Amenities needed to safely enjoy parks	Six categories: recreational, information, educational, accommodations, parking, other
What kind of natural place do you usually visit? (select all options that apply)	Type of natural location usually visited	Eight categories: beach, lake, river, cottage, wetland, forest, mountain, other
What is your favorite landscape? (select all options that apply)	Favorite landscape	Seven categories: ocean, lake, agricultural, wetland, forest, mountain
How do you prepare for a trip to a natural place?	Information search tool to prepare a visit to a natural area	Eight categories: friend’s recommendation, knows area, social networks, Google search, official website, travel agency, guidebook Clustered into four categories: knowledge, random internet search, website, and traditional tool
What is your principal barrier regarding paths in natural areas? (select all options that apply)	Path’s principal barrier	Six categories: length, distance from visitor center, slope, pavement, weather, other
How often do you visit a national park?	NPs visitation frequency	Seven categories: Never, one every few years, once a year, a few times a year, at least once a month, at least once a week, every day Clustered into four categories: never, seldom, yearly, often
In the last year, have you enjoyed at least one of these natural places: beach, lake, river, farmland, wetland, forest/vegetable garden, mountain, other	Frequency of visits to natural locations in the last year	Five categories: never, few times a year, at least once a month, at least once a week, every day Clustered into three categories: never, occasionally, often
How do you prepare for a trip to a natural place?	Trip planning	Eight categories: official website, random Google search, travel agency, guidebook, social networks, knows area, other Clustered into four categories: official website, random Google search, knowledge, and traditional sources

### 3.2. Statistical analysis

A descriptive analysis with Jamovi Project (2021) was done to gain an overview of their needs and conditions that affect their decision “to go to parks,” and their nature-based-tourism experience challenges when on site. Some of the categories in the variables were clustered to be able to properly work statistically with the sample obtained (Tables 1, 2; Cea and Ancona, 2001).

Then, log-linear models were used to analyze the relationship, assessing associations and interactions, between two or more categorical variables by modeling their cross-frequency table (Fagerland et al., 2017; Agresti, 2018). The model is represented by the expected frequencies of the cross-frequency table as a function of parameters representing the effects of the categorical variables using a Poisson linear model (Lang, 2005). The effects of the variables (or associations between variables) are described in terms of the log of the odds of the joint probability distribution of the variables, that is, in terms of the log of the odds of the events of the cross-frequency table. The odds (i.e., the exponential of the log of the odds) of a single event in the cross-frequency table:


$$l\,o\,g\,\left(o\,d\,d\,s_{A}\right)=l\,o\,g\,\left(\frac{\pi_{A}}{1-\pi_{A}}\right)$$

that is, the log of the odds of event A of the cross-frequency table is the log of the ratio between the probability of that event occurring and the probability of that event not occurring. The odds (i.e., the exponential of the log of the odds) of an event in the cross-frequency table represents the probability of that event occurring relative to its not occurring. The log of the odds of an event A relative to any other event B can be computed by subtracting their log of the odds.


(1) $$l\,o\,g\,\left(\frac{o\,d\,d\,s_{A}}{o\,d\,d\,s_{B}}\right)=l\,o\,g\,\left(o\,d\,d\,s_{A}\right)-l\,o\,g\,\left(o\,d\,d\,s_{B}\right)$$

Then, the probability of one event relative to another is obtained by exponentiating the corresponding relative log of odds. The R software and the “glm” R function (R Core Team, 2020) are used to apply the log-linear models to the data.

## 4. Results

After ensuring the database’s adequacy, removing people who did not complete the entire questionnaire and minors from the total people who participated in the survey, the valid sample statistically analyzed was *n* = 118. Initially, a descriptive analysis was used to explore the demographic characteristics of the data. Then Pearson’s chi-square test was applied to check the independence of the public use of green areas and the type of device needed. Finally, log-linear statistical models were applied to assess the associations and interactions between the variables involved in each of the hypotheses of the study, which aimed to determine which aspects limit PwMDs’ public use of national parks (NPs).

### 4.1. Descriptive analysis

Univariate analysis was applied to explore how the variables behave and to provide us with a general understanding of the relationship between PwMD characteristics and their behavior related to enjoying nature. Descriptive analysis from the total sample *n* = 118 shows for demographic population characteristics (Table 3) higher participation in Spain than in Canada (60–40%, respectively). Regarding age groups, the highest participation was among adults (70%), with notably low participation of young adults (6%). About assistance device type, wheeled mobility devices (74%) are the most representative sample. Finally, most of the respondents had driver’s licenses (76%) and their own car (74%). Regarding “*preferred landscape*” and “*greener area*” usually visited, results revealed the desired landscapes are mountains, forests, and the ocean (32, 29, and 26%) while beaches, forests, and mountains were the natural location visited the most often (29, 23, and 17%).

**TABLE 3:** Descriptive results from PwMDs demographic population characteristics data frequencies.

	Relative frequency %
**Demographic characteristics**	
**Location (%)**	
Canada	40
Spain	60
**Gender (%)**	
Female	40
Male	58
Other	2
Prefer not to say	2
**Age groups (%)**	
Young adults	6
Adults	70
Older adults	24
Prefer not to say	1
**Assistance device (%)**	
Crutches	7
Walkers	4
Prosthesis	2
Walking stick/canes	14
Manual wheelchair	51
Power wheelchair	20
Mobility scooters	3
**Assistance device clustered (%)**	
Ambulation aids	26
Wheeled mobility devices	74
**Driver’s license (%)**	
Yes	76
No	24
**Own car (%)**	
Yes	74
No	26
**Green areas most visited (%)**	
Beach	29
Lake	11
River	9
Cottage/farmland	5
Wetland	4
Forest	23
Mountain	17
**Other**	1
**Landscape preferences (%)**	
Mountain	32
Ocean	26
Meadow	7
Forest	29
Wetland	3
Agricultural	1
Other	2

Bivariate analysis (Table 4), through the Pearson’s chi-square test for section 2 of the questionnaire, relating public use of parks with their assistance device type (wheeled mobility devices, and ambulation aids) shows for most of the variables independence of the assistance device type in terms of public use, confirmed by *p*-value > 0.05. Results revealed that PwMDs like to enjoy nature regardless of the assistance device type (100% who use ambulation aids and 99% who use wheeled mobility devices). However, the *frequency category* related to visiting NPs most chosen is “*Once every few years*” (31 and 21%, respectively). There is a general consensus that they would like to enjoy it more often (97 and 90%, respectively). When asked about their enjoyment of nature in greener areas usually related to leisure time such as the beach, rivers, and cottages, most of those surveyed responded: “*few times per year*” (54 and 48%, respectively). The next question focuses on people who chose “never.” There is an inconsistency [**χ ^2^=** 14 (H_0_ > 0 of independency)]; *p*-value = 0.03 (*p*-value < 0.05 of dependency) because all of those surveyed answered the question. Although the question was intended only for people who do not enjoy green areas, most of the surveyors answered it. Their responses are interesting because inaccessibility is highlighted as the main reason (39 and 21%). The lack of information about accessibility (13 and 10%) is also selected. Finally, the official website is the tool most used to search for information about natural areas (59 and 47%, respectively).

**TABLE 4:** Results of Pearson’s chi-square test of the first part of questions in section II on public nature use.

	Frequency		Pearson χ^2^ test	
	Wheeled mobility devices *n* = 74%	Ambulation aids *n* = 26%	χ^2^	*P*-value
**Like to enjoy nature**				
Like	99%	100%	0.329	0.566
Dislike	1%	0%		
**Frequency of visits to NP**				
Never	19%	38%	6.12	0.295
Once every few years	31%	21%		
Once per year	15%	14%		
Few times per year	28%	28%		
At least once a month	4%	0%		
At least once a week	2%	0%		
Every day	0%	0%		
**Visit NP more often**				
Yes	97%	90%	2.2	0.138
No	3%	10%		
**Frequency visits to green areas last year**				
Never	13%	14%	1.42	0.841
Few times per year	54%	48%		
At least once a month	15%	10%		
At least once a week	16%	24%		
Every day	2%	3%		
**The main reason to never go to green areas**				
They did not respond “never”	30%	38%	14	0.03
Natural places are inaccessible to me	39%	21%		
The distance to those areas is too great	6%	0%		
I don’t feel welcome in these areas	4%	3%		
I don’t have enough information about these areas	13%	10%		
I don’t have my own means of transportation	2%	17%		
Other	4%	10%		
**NP visits in home country**				
Empty	4%	0%	1.74	0.42
Yes	78%	86%		
No	18%	14%		
**NP visits in other than home country**				
Empty	4%	0%	2.54	0.469
Yes	8%	14%		
No	69%	62%		
Text name if you remember it	19%	24%		
**Information searching tools to prepare park visit**				
Social networks	10%	3%	7.73	0.366
Friend recommendation	6%	14%		
Google search	22%	7%		
Guide book	1%	3%		
Knowing area	10%	10%		
No response	1%	0%		
Official website	47%	59%		
Travel agency	2%	3%		

In the following section, log-linear models are applied to explore specific aspects of the outdoor experience in NPs in more depth based on the hypothesis formulated to design the questionnaire.

### 4.2. Log-linear model analysis

The relationships between questions/variables considered in the questionnaire and organized according to the hypotheses formulated in the study are analyzed through log-linear models that allow estimating the associations between the questions/variables in terms of the odds of the joint probability distribution. For each relationship analyzed, the log of the odds of the events of the corresponding cross-frequency table is represented. As commented in section “3.2 Statistical analysisl above, the log of the odds of an event relative to any other event can be computed by subtracting their log of the odds (Equation 1). Then, the probability of one event relative to another (i.e., the odds of an event relative to another) is obtained by exponentiating the corresponding relative log of odds.

The 95% confidence intervals of the log-odds estimates are also obtained. The graphical assessment of overlap across confidence intervals is used to assess the significance of the probability of one event relative to another, as well as the trend of probabilities between events.

#### 4.2.1. Visit preparation and demographic characteristics of PwMDs (hypothesis 1)

The analysis of PwMDs’ use of different information search tools to prepare for their visit and its relationship with the demographic and individual characteristics such as country, gender, age, mobility assistive devices, driver’s license, and own car is included here.

**FIGURE 1:**
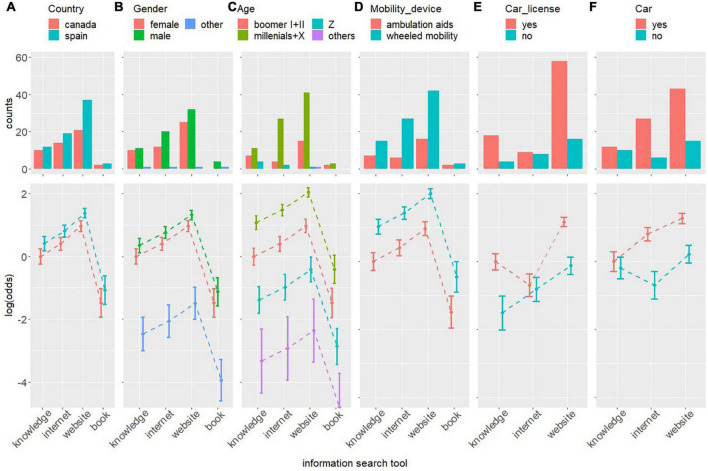
Histograms and the logarithm of the odds resulting from applying a log-linear model in the cross-frequency tables of the relationships between the demographic and individual characteristics of, from left to the right, country **(A)**, gender **(B)**, age **(C)**, mobility assistive device **(D)**, driver’s license **(E)**, and own car **(F)** of PwMDs and PwMDs’ use of different information search tools to prepare the visit.

The histograms of the cross-frequency tables between the use of the different information search tools and the demographic and individual characteristics of PwMDs are depicted in the top row of Figure 1. The mean and 95% confidence intervals of the log of the odds ratio resulting from applying the log-linear model in the cross-frequency tables of each of the relationships are depicted in the bottom row of Figure 1.

People with mobility/motor disabilities’ use of the different information search tools are independent of the demographic and individual characteristics of the country, gender, age, and mobility assistive devices of PwMDs, where the most likely information search tool used by PwMDs is “official website,” followed by “random internet search,” “prior knowledge,” and, finally, “traditional tools.” Thus, the probability of using the “random internet search” is approximately 1.5 times greater than using “prior knowledge,” as their relative log of the odds is 0.405 [i.e., exp (0.405)≈1.5]; the probability of using the “official website” is approximately 1.75 times greater than using “random internet search,” as their relative log of the odds is 0.564 [i.e., exp (0.564)≈1.75]; and the probability of using “traditional tools” is approximately 1/12 that of using the “official website” information search tool, as their relative log of the odds is −2.451 [i.e., exp (−2.451)≈1/12].

People with mobility/motor disabilities’ use of the different information search tools is not independent of whether the PwMDs have a driver’s license or their own car. When they do not have a driver’s license, the most likely information search tool used by PwMDs is “official website,” followed by “random internet search” and “prior knowledge,” where the probability of using “random internet search” is approximately two times greater than using “prior knowledge,” as their relative log of the odds is 0.693, and using the “official website” is also approximately two times greater than the “random internet search,” as their relative log of the odds is 0.694. However, when they do have a driver’s license the probability of using “random internet search” decreases significantly, at approximately 1/7 of that of the “official website,” as their relative log of the odds is −1.863, and ½ of that of “prior knowledge,” as their relative log of the odds is −0.693.

When they have their own car, the most likely information search tool used by PwMDs is “official website,” followed by “random internet search” and “prior knowledge,” where the probability of using a “random internet search” is approximately 2.1 times greater than using “prior knowledge,” as their relative log of the odds is 0.811, and using the “official website” is also approximately 1.6 times greater than using a “random internet search,” as their relative log of the odds is 0.465. However, when they do not have their own car the difference among the probabilities of the different information search tools is not as pronounced, and the probability of using “random internet search” is even lower than that of “prior knowledge.”

In summary, in general, the most likely information search tool used by PwMDs is “official website,” followed by “random internet search,” “prior knowledge” and, finally, “traditional tools.” However, when PwMDs have a driver’s license, the probability of using the “random internet search” is significantly lower than that of using “prior knowledge,” and when they do not have their own car the probabilities of the different information search tools are not as different, although “website” is still greater than the others.

#### 4.2.2. Perception of park barriers and frequency of visits (hypothesis 2)

The analysis of the perception of the distance traveled, the distance from the visitor center, the slope, the type of pavement, and the weather as barriers for PwMDs to visit and enjoy natural sites, and whether perceiving them as barriers or not might depend on PwMDs’ usual visit frequency to these sites is included here.

**FIGURE 2:**
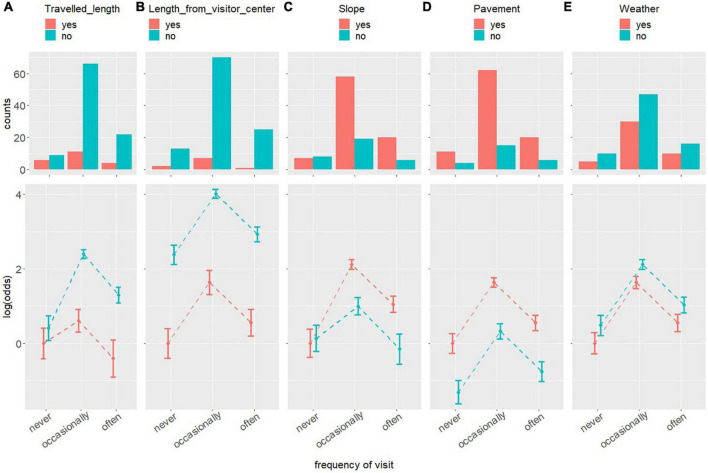
Histograms and the logarithm of the odds resulting from applying a log-linear model in the cross-frequency tables of the relationships between the perception of, from left to the right, the distance traveled **(A)**, the distance from the visitor center **(B)**, the slope **(C)**, the type of pavement **(D)**, and the weather **(E)** as barriers for PwMDs to visit and enjoy natural sites and PwMDs’ visit frequency to these sites.

The histograms of the cross-frequency tables between the frequency of visits and the perception of each of the barriers are depicted in the top row of Figure 1. The mean and 95% confidence intervals of the log of the odds ratio resulting from applying the log-linear model in the cross-frequency tables of each of the relationships are depicted in the bottom row of Figure 2.

The perception of the distance from the visitor center, pavement, and weather as barriers for PwMDs are independent of the frequency of visits, and vice versa, i.e., the frequency of visits is independent of the perception of the distance from the visitor center, pavement, and weather as barriers for PwMDs. The most likely frequency of visits is “occasionally,” followed by “often,” and finally “never,” and the frequency of visits of “occasionally” is approximately 5.1 times greater than that of “never,” as their relative log of the odds is 1.636, and 3 times greater than that of “often,” as their relative log of the odds is 1.086, and that of “often” is approximately 1.7 times greater than that of “never,” as their relative log of the odds is 1.733. All these differences are statistically significant.

Participants are more likely to not perceive the distance from the visitor center as a barrier than they are to perceive it as one, as the log of the odds of not perceiving it is 2.37, which means that the probability of not perceiving it as a barrier is approximately 10.8 times greater than of perceiving it.

Participants are more likely to perceive the pavement as a barrier than they are to not, as the log of the odds of not perceiving it is −1.31, which means that the probability of not perceiving it as a barrier is approximately 1/3.8 of that of perceiving it, or, to put it the other way around, the probability of perceiving it as a barrier is approximately 3.8 times greater than that of not perceiving it as a barrier.

Participants are more likely to not perceive the weather as a barrier than they are to perceive it as one, as the log of the odds of not perceiving it is 0.48, which means that the probability of not perceiving it as a barrier is approximately 1.61 times greater than perceiving it as one.

Participants are more likely to not perceive the distance traveled as a barrier than they are to perceive it as one, as the log of the odds of not perceiving it for all the visit frequencies is greater than 0, which means that the probability of not perceiving it is greater than that of perceiving it. The perception of the distance traveled as a barrier is mostly independent of the frequency of visits, and vice versa, with the exception of the frequency of visits of “never” where the difference between perceiving it and not perceiving it as a barrier is not statistically significant. The difference between the log of the odds of not perceiving it as a barrier and that of perceiving it at the frequencies of the visit of “occasionally” and “often” are 1.81 and 1.68, respectively, which means that the probabilities of not perceiving it as a barrier are approximately 6.1 and 5.3 times greater than that of perceiving it, respectively. When the distance traveled is not perceived as a barrier, the most likely frequency of visit is “occasionally,” followed by “often,” and finally “never,” where the frequency of visit of “occasionally” is approximately 6.7 times greater than that of “never,” and approximately 2.7 times greater than that of “often.” A similar pattern is found when the distance traveled is perceived as a barrier, but the difference between the frequency of visits of “occasionally” and “often” is the only one that is statistically significant, where the frequency of visits “often” is 2.4 times lower than that of “occasionally.”

Participants are more likely to perceive slope as a barrier than they are to not, as the log of the odds of perceiving it is greater than 0, except at a frequency of visit of “never,” where the difference between perceiving it and not perceiving it as a barrier is not statistically significant. The difference between the log of the odds of perceiving it as a barrier and of not perceiving it at the frequencies of the visit of “occasionally” and “often” are 1.2 and 1.3, respectively, which means that the probabilities of perceiving it as a barrier are approximately 3.4 and 3.7 times greater than that of not perceiving it, respectively. When perceiving the slope as a barrier, the frequency of visits of “occasionally” is approximately 9 times greater than that of “never,” and approximately 2.8 times greater than that of “often.” When the slope is not perceived as a barrier, the frequency of visits of “occasionally” is approximately 2.2 times greater than “never,” and approximately 3.3 times greater than that of “often.”

In short, the probabilities of perceiving or not perceiving the features as barriers are mostly independent of the frequency of visits, with the exception of the frequency of “never” for the barriers of distance traveled and slope. The pavement and the slope are more likely to be perceived as barriers than not perceived as barriers, with a probability of perceiving them as such around 3–4 times greater, on average than that of not perceiving them as barriers. The distance traveled, the distance from the visitor center, and the weather are more likely to not be perceived as barriers than perceived as barriers, with probabilities of approximately 5, 10, and 1.6 times greater of not perceiving them as barriers, on average, than of not perceiving them as barriers, respectively. The most likely frequency of visit is “occasionally,” followed by “often,” and finally “never” for all of the barriers with the exception of slope and distance traveled in which there are no statistically significant differences between the frequencies of the visit of “never” and “often.”

#### 4.2.3. Amenities needed and frequency of visit (hypothesis 3)

The analysis of the requirement (or not) of recreation (footpath, picnic area, lookout, wildlife-hide), information (signposting), educational (visitor center, museum, information point, botanical garden), accommodations (camping, hostel, hotel, or other options to stay overnight), and parking as essential amenities to PwMDs to visit and enjoy natural sites, and whether requiring them or not might depend on PwMDs’ usual visit frequency to these sites is included here.

**FIGURE 3:**
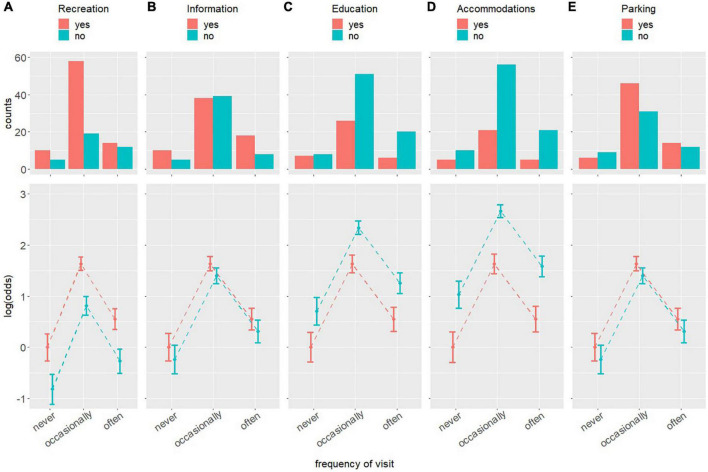
Histograms and the logarithm of the odds resulting from applying a log-linear model in the cross-frequency tables of the relationships between the need of, from left to the right, recreation **(A)**, information **(B)**, education **(C)**, accommodations **(D)**, and parking **(E)** as amenities for PwNDs to visit and enjoy natural sites and PwNDs’ visit frequency to these sites.

The histograms of the cross-frequency tables between the frequency of visits and each of the amenities required by PwMDs are depicted in the top row of Figure 3. The mean and 95% confidence intervals of the log of the odds ratio resulting from applying the log-linear model in the cross-frequency tables of each of the relationships are depicted in the bottom row of Figure 3.

The requirement or not a requirement of the amenities by the PwMDs to visit and enjoy natural sites are independent of the frequency of visit, and vice versa, i.e., the frequency of visit is independent of PwMDs’ requiring it or not. The most likely frequency of visit is “occasionally,” followed by “often,” and finally “never,” where the frequency of visit “occasionally” is approximately 5.1 times greater than that of “never,” as their relative log of the odds is 1.636, and 3 times greater than that of “often,” as their relative log of the odds is 1.086, and that of “often” is approximately 1.7 times greater than that of “never,” as their relative log of the odds is 1.733. All these differences are statistically significant.

Participants are more likely to require recreation as an essential amenity than they are to not, as the log of the odds of requiring it is 0.82, which means that the probability of requiring it as an essential amenity is approximately 2.3 times greater than that of not requiring it.

Participants are slightly more likely to require information and parking as essential amenities than they are to not, although these differences cannot be said to be statistically significant, as the uncertainties in the log of the odds of requiring and not requiring them to overlap each other significantly in both information and parking amenities.

Participants are more likely to not require education and accommodations as essential amenities than they are to require them, as the log of the odds of not requiring them are 0.70 and 1.03, respectively, which means that the probabilities of not requiring them as essential amenities are approximately 2 and 2.8 times greater, respectively, than that of requiring them.

In short, the probabilities of requiring or not requiring the amenities to visit and enjoy natural sites are independent of the frequency of visits. PwMDs are likely to require recreation as an essential amenity and are not likely to require education and accommodations as essential amenities. PwMDs tend to require information and parking as essential amenities, but this cannot be stated in a statistically significant way. The most likely frequency of visit is “sometimes,” followed by “high,” and finally “never.”

#### 4.2.4. PwMDs’ visited vs. preferred natural locations (hypothesis 4)

The analysis of several natural sites, such as beaches, lakes, rivers, cottages, wetlands, forests, and mountains is included here. The analysis is focused on evaluating the existence of a relationship between whether or not they are desired places and whether or not they are usually visited. The comparison of PwMDs’ preferences or desires with the places actually traveled to by PwMDs can allow us to evaluate if they are able to enjoy those places that they really want to or whether they only enjoy the places that *a priori* may be more suitable for them due to their physical conditions.

**FIGURE 4:**
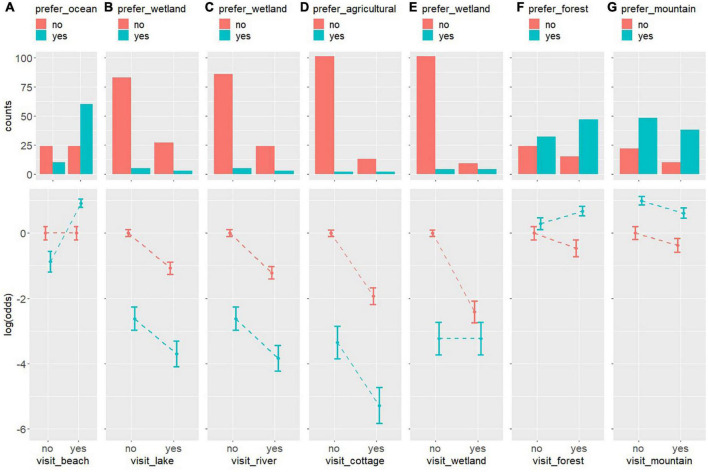
Histograms and the logarithm of the odds resulting from applying a log-linear model in the cross-frequency tables of the relationships between landscape type preference and green location type usually visited by PwMD, from left to right, ocean vs. beach **(A)**, wetland vs. lake **(B)**, wetland vs. river **(C)**, agricultural vs. cottage **(D)**, wetland vs. wetland **(E)**, forest vs. forest **(F)**, mountain vs. mountain **(G)**.

The histograms of the cross-frequency tables between preference and PwMDs’ actual visits to the natural sites are depicted in the top row of Figure 4. The mean and 95% confidence intervals of the log of the odds ratio resulting from applying the log-linear model in the cross-frequency tables of each of the relationships are depicted in the bottom row of Figure 4.

Visits to the beach or not are related to preference for the ocean. When PwMDs prefer the ocean, the probability of visiting the beach is approximately 4.5 times greater than not visiting it, since the log of the odds of visiting the beach is 1.5. However, when there is no PwMD preference for the ocean, there is no difference between the probability of visiting and not visiting the beach.

Visiting lakes or rivers or not is not related to the preference or not for wetlands. The probability of PwMDs not visiting lakes and rivers is approximately 2.7 and 3.3 times higher, respectively, that visiting them, regardless of whether or not they show a preference for wetlands. Most PwMDs showed a preference for wetlands.

Visiting cottages is not related to the preference or not for agriculture. The probability of PwMDs not visiting cottages is approximately 6.8 times higher than visiting them, regardless of whether or not they show a preference for agriculture. Most PwMDs showed a preference for agriculture.

People with mobility/motor disabilities visiting wetlands or not is related to whether or not they show a preference for it. When PwMDs’ do not have a preference for wetlands, the probability of not visiting them is approximately 11 times greater than visiting them, since the log of the odds of not visiting them when they do not show a preference is 2.4. However, when PwMDs prefer wetlands, there is no difference between the probability of visiting and not visiting them.

People with mobility/motor disabilities visiting forests or not is related to whether or not they show a preference for it. When PwMDs prefer forests, the probability of visiting them is approximately 1.4 times greater than not visiting them, since the log of the odds of visiting it when they do show a preference is 0.35. However, when PwMDs do not prefer forests, the probability of not visiting it is approximately 1.6 times greater than visiting it, since the log of the odds of not visiting it when they do not show a preference is 0.47.

People with mobility/motor disabilities visiting mountains or not is related to whether or not they show a preference for it. The probability of not visiting the mountain by PwMD is approximately 1.45 times higher than visiting it, respectively, regardless of whether or not they show a preference for it. Most PwMDs showed a preference for mountains.

## 5. Discussion

The results from the descriptive analysis confirm that PwMD likes to enjoy nature, and, moreover, that they would like to enjoy it more often than they can as other authors have confirmed (Williams et al., 2004; Burns and Grafe, 2007; Stigsdotter et al., 2017; Zhang et al., 2017; Corazon et al., 2019; Blaszczyk et al., 2020; Perry et al., 2021). Despite this evidence, doing so is still very difficult due to the multiple barriers that the group has to face to enjoy natural spaces equitably (Burns et al., 2009; Corazon et al., 2019; Menzies et al., 2020) and benefit from their ecosystem services (Zhang et al., 2017; Groulx et al., 2021).

There are some differences regarding the frequency with which people who use ambulation aids and people who use wheeled mobility devices visit nature (Mahmood et al., 2019). The first group goes to NPs less frequently. This difference may be attributed to the fact that most of the respondents belong to the second group or because they have difficulties walking longer distances in outdoor areas like NP. Nevertheless, both groups agree that they would like to enjoy NPs more often if outdoor conditions constrained them less due to their physical conditions, which some authors (Williams et al., 2004; Burns et al., 2009; Menzies et al., 2020) have also confirmed.

Internet and communication technologies (ICTs) are the most used tools to obtain park accessibility information for PwMDs to learn about park accessibility conditions before going, and the *official park website* is the one they are most likely to check, independently of their demographic background. Results show the importance of NPs having an up-to-date official website with accurate information on accessibility for PwMD and on what activities they can participate in Tsai et al. (2010), Aguilar-Carrasco et al. (2017), Zhang et al. (2017), Corazon et al. (2019), and Bianchi et al. (2020). Additionally, results show there is a relationship between the information tool used and whether they have a *driver’s license* and their *own car* or not, and here the *official website* is the source most used as well. This could be due to the fact that having a vehicle makes it easier for them to reach the more remote areas where NPs are usually located (Burns and Grafe, 2007; James et al., 2017; Menzies et al., 2020). Also, if they know in advance where to park their vehicle in the vicinity of the park or of the paths that run through it, information parks management should provide, their visit would be easier since parking is one of the barriers cited in previous studies (Williams et al., 2004; Burns and Grafe, 2007; Zhang et al., 2017), as well as the lack of accessible public transport.

Regarding structural constraints, perceived barriers are mostly independent of the frequency with which they visit nature. *Pavement* and *slope* were chosen as structural constraints while *distance traveled*, the *distance from the visitor center*, and the *weather* was more likely to not be perceived as barriers than perceived as them, in agreement with other studies (Zhang et al., 2017; Corazon et al., 2019). These two barriers can be addressed with adequate planning based on the different intersectional realities, accompanied by adequate maintenance, in short, by good governance (Aguilar-Carrasco et al., 2022). Taking into consideration that “*occasionally*” is the most likely frequency, it seems there is something more than physical constraints since there is not a statically significant difference between PwMD who *never* or *often* go to any natural location. Among these PwMDs, *distance traveled* and *slope* were chosen as barriers that might keep them from equally enjoying nature. It could be due to intrapersonal and interpersonal constraints on those who never enjoy nature, or on those who although they are aware of the difficulty, they go often anyway because they can move along paths with these barriers, either because their physical level allows them to do so or because they have logistical support and the right devices (Williams et al., 2004; Burns and Grafe, 2007; Stigsdotter et al., 2017).

*Recreation* is seen as the most essential amenity independent of the frequency with which they visit nature. This clustered all devices which can keep them from wheeling or walking within the park, such as footpaths, picnic areas, lookouts, and wildlife blinds. For that reason, the amenities do not seem to be the main constraints associated with the frequency of visiting natural spaces, as in Stigsdotter et al. (2017). However, an important aspect of a successful experience (Corazon et al., 2019) as amenities could have an impact on individual (intrapersonal) psychology and other interpersonal factors that allow them to go outdoors (Williams et al., 2004; Zhang et al., 2017). Similar results can be seen with *information* and *parking* which tend to be required as essential amenities, but this cannot be stated in a statistically significant way. The latter is noteworthy because in the literature reviewed parking has been highlighted as an issue so we expected this would be an important amenity to them, especially to enjoy remote areas such as NPs (Burns and Grafe, 2007).

The most likely frequency of visiting parks is *occasionally*, followed by *often*, and finally *never*, the same result as when the frequency of visits is intersected with barriers as explained above. The frequency of visits to natural spaces seems to be associated with physical factors of the environment that keep PwMD from enjoying the outdoors, but at the same time, it seems that intrapersonal and interpersonal factors play an important role in their decision. For this reason, according to Groulx et al. (2021), all efforts done by parks administration in terms of management should be supported by working hand-in-hand with PwMDs to address interpersonal and intrapersonal factors.

Finally, we would like to shed light on the question of whether PwMD is able to enjoy their preferred landscapes or if they only enjoy a few suitable places due to their physical conditions (Williams et al., 2004; Stigsdotter et al., 2017). Our results confirmed in some way that they are not enjoying those preferred areas more often, for example, visiting or not the *beach* is not related to preferring the *ocean* as a landscape. Beaches are a tourist resource widely used by people on vacation and in the summer, so it is a natural asset desired by all, and as a tourist resource, it is slowly being better adapted to the needs of the PwMD, as shown by the Spanish case evaluated in Mayordomo-Martínez et al. (2019). Interesting results were obtained when *forest* landscape with *forest* areas intersected, which shows that visiting them is related to whether they show a preference for these natural locations, confirming the findings of Corazon et al. (2019) that PwMD would like to enjoy the forest more often than they can and would if those areas were more suitable for them. On the contrary, despite what we initially thought, in the case of visiting *mountains*, it was not demonstrated that it is directly related to a preference for those landscapes. The *mountains* could be *a priori* difficult location for PwMD’s physical condition, thus, this result confirms that PwMD is more likely to enjoy outdoor activities which are less physically demanding although they would love to go there. Moreover, it should be taken into consideration that those activities in rugged places might require major adaptations for PwMD walkability or wheelability, which at the same time could alter the landscape that is not desired for PwMD (Burns and Grafe, 2007; James et al., 2017; Stigsdotter et al., 2017; Zhang et al., 2017; Corazon et al., 2019; Menzies et al., 2020; Groulx et al., 2021, 2022).

## 6. Conclusion

Participants with motor/mobility disabilities occasionally visit green spaces, although these are in populated areas or in remote places such as NPs. The results obtained from the frequency of visits crossed with the proposed barriers and amenities confirm that physical environment constraints are not the only aspect that is clearly discouraging them from going to parks more often. Of the environmental barriers, slopes and pavement on paths are factors that limit the ability of people with mobility or motor disabilities to go into forests or enjoy other outdoor options. They have preferences for outdoor spaces such as forests or mountains, while most of the participants more often enjoy natural locations which are usually anthropized like urban beaches. The most recurrent source of information to learn about outdoor accessibility in advance is the site’s official website.

The results of this study shed light on the frequency of visits to NPs and some of their physical environmental limitations. It has also revealed how natural spaces should be equipped and what barriers in the park’s paths prevent them from being walkable/wheelable to allow for a more rewarding wilderness experience. There are still issues that limit them, such as interpersonal and intrapersonal relationships, with the environment that an online survey model does not seem to be the best system to address. Clearly, more research is required on these constraints faced by PwMD. In addition, given the consensus in the reviewed literature that people with motor/mobility disabilities want to have full and enriching experiences in natural spaces, without causing irreparable damage to the NP or incurring any loss of naturalness.

It is worth noting that one of the limitations of the study concerns the datasets that only provide one small picture of the universe population of PwMD in both countries. However, the focus of this study was to explore possible physical barriers based on the literature review. It is evident that more research is required, and it could be focused on two more fields: first, as the results obtained show that there are more than only physical barriers, future studies should explore psychological barriers further through research that involves the active participation of PwMD in parks. Also, in terms of park management future studies should address aspects related to the intrinsic accessibility for PwD of natural areas such as NPs taking into consideration the physical environment as well as interpersonal and intrapersonal constraints to propose comprehensive solutions that allow PwD to also enjoy nature-based recreation at parks.

## Data availability statement

The raw data supporting the conclusions of this article will be made available by the authors, without undue reservation.

## Ethics statement

The study was approved by the Behavioural Research Ethics Board of the University of British Columbia (Approval Certificate Number: H190-00951). Written informed consent for participation was not required for this study in accordance with the national legislation and the institutional requirements.

## Author contributions

MJA-C contributed to the conceptualization and questionnaire design, drafted the application for the H190-00951 certificate, and monitored it through to approval and formal analysis, research, methodology, results, discussion, and writing and editing of the original draft. EG, MV-P, and FG contributed to writing and editing the original draft. GR-M designed the methodology of statistical modeling and analysis and contributed to writing and editing the original draft. All authors contributed to the reading and have agreed to the published version of the manuscript.
